# The combined effects of artificial gravity, temperature, and hypoxia on haemodynamic responses and limb blood flow

**DOI:** 10.1007/s00421-025-05773-7

**Published:** 2025-04-02

**Authors:** Jason T. Fisher, Urša Ciuha, Pierre Denise, Adam C. McDonnell, Hervé Normand, Igor B. Mekjavic

**Affiliations:** 1 Department of Automatics, Biocybernetics and Robotics, Jožef Stefan Institute, Jamova 39, 1000 Ljubljana, Slovenia; 2 Jožef Stefan International Postgraduate School, Jamova 39, 1000 Ljubljana, Slovenia; 3 Université de Caen Normandie, Inserm, Cyceron, CHU de Caen, COMETE U1075 Caen, France

**Keywords:** Cardiovascular, Microvascular blood flow, Artificial gravity, Hypoxia, Thermoregulation, Baroreflex

## Abstract

Under simultaneous environmental and gravitational stressors, integrated vascular responses maintain homeostatic balance via coordinated baro- and thermo-regulatory action. The effect of temperature and hypoxia at an elevated gravitational vector on the interaction of these systems was examined. Ten male participants experienced either cool (18.4 °C) or warm (29.1 °C) ambient temperatures in normoxia (partial pressure of oxygen, P_I_O_2_ = 133 mmHg) or hypoxia (P_I_O_2_ = 92 mmHg). Cardiovascular (heart rate, HR; arterial pressure, MAP; cardiac output, CO; stroke volume, SV; skin blood flow, SkBF) and thermoregulatory (skin temperature; core temperature) responses were monitored during standing (NG), and supine centrifugation at ground reaction forces (GRF) measured with a force platform at 1GRF and 2GRF. At 2GRF, warm and hypoxic conditions reduced the test duration by 16%. No differences were observed between NG and 1GRF in any variable; however, 2GRF significantly raised HR by 29.3% and MAP by 12.6%, and lowered SV by 22.2%. Warm condition significantly increased HR, and significantly decreased MAP and SV compared to the cool condition, by 17.8%, 6.1%, and 5.8%, respectively. Hypoxia had no effect on any variable. Arm SkBF significantly decreased by 33.3% with increasing artificial gravity, whereas leg SkBF increased by 38.7%. Higher ambient temperatures had no effect on leg SkBF, but significantly increased arm SkBF by 38.7%. Human tolerance to passive centrifugation is significantly lower at 2GRF, and further affected by the ambient conditions. Haemodynamic and leg SkBF responses in higher temperature and Gz conditions were frequently unable to prevent pre-syncopal symptoms. Finally, arm SkBF was modulated by both baroreflex and thermoregulation, and the baroreflex alone in leg SkBF.

## Introduction

Changes in the hydrostatic pressure gradient within the circulation can compromise the regulation of arterial blood pressure. The ability to withstand these changes is defined as orthostatic tolerance (OT). A multitude of factors contribute to OT, including body (and ambient) temperature, acute or chronic hypotension, physical fitness, hydration, and anthropometrics (Brignole 2007; Christou and Kiortsis 2017; Levine et al. 1991; Stewart 2013; Wilson et al. 2006). The role of the blood pressure regulatory system is to maintain appropriate perfusion of vital organs, particularly the central nervous system (CNS), by preventing pooling of blood in the lower extremities during exposure to natural gravity in the upright posture. The head-to-foot gravitational vector can also be artificially elevated above 1 Gz, in which case the ability to maintain arterial pressure under such conditions is termed gravitational, or passive G-tolerance. Studies have shown that the baroreflex is capable of maintaining adequate blood pressure in individuals even at suitably high g-loads, known as G-tolerance. Goswami et al. (2015) proposed that centrifugation of subjects in a supine position on a short-arm human centrifuge (SAHC) with 0.75 Gz at heart level represents similar cerebrovascular and cardiovascular responses as observed in the upright standing position, at an ambient temperature of 22 °C. Above these levels of G-load, the increased gravitational stress may lead to a detrimental decrease in cerebral perfusion pressure, and thus cerebral blood flow (Ogawa et al. 2016), eliciting syncope with increasing Gz or prolonged exposure. Despite the potential for syncopal symptoms, the use of SAHC as a potential training tool for astronauts, to assist in the maintenance of a suitable arterial baroreflex operational range and subsequent OT (Adami et al. 2013), is of significant interest.

Concurrent with blood pressure control, regulation of homeothermy in the face of ranging ambient temperatures (Ta) is achieved via autonomic responses, in addition to behavioural responses. Above and below the so-called ‘critical temperatures’ of Ta, mechanisms of heat loss (upper critical temperature) and production (lower critical temperature) initiate sweat secretion and shivering thermogenesis, respectively. However, within these upper and lower critical temperatures exists the ‘vasomotor zone’ (Mekjavic et al. 1991), whereby sympathetic neural control mechanisms produce vasodilatory and vasoconstrictor vascular control. Within this vasomotor zone, skin blood flow (SkBF) can vary from as little as 0.25 L·min^−1^ to as much as 6–8 L·min^−1^ (Charkoudian 2003).

The chemoreflex also influences regional vascular control, which is activated under hypoxic conditions, and may alter OT responses. Hypoxaemia elicits peripheral vasodilatation and increases skeletal muscle blood flow despite an increase in sympathetic activity (Dinenno 2016) originating from the stimulation of α-adrenergic receptors on vascular smooth muscle (Jacob et al. 2021). There is a complex interaction between the sympathoadrenal system and locally derived vasodilatory substances (including NO) that ultimately determine the net peripheral vasodilatory response to systemic hypoxia in humans. Whereas the effect on muscular blood flow appears to be uniform throughout the body, the effect of hypoxia on SkBF is less uniform, seemingly with an increase in peripheral non-acral SkBF, and a decrease in peripheral acral SkBF and central SkBF (Golja and Mekjavić, 2002; Minson 2003).

At lower G-loads, it is clear that the baroreflex is capable of maintaining arterial pressure, with the magnitude of vascular response increasing with increasing G-load. It has previously been shown, however, that the influence of thermoregulation for the maintenance of homeothermy during gravitationally challenging situations creates interaction (or competition) in autonomic control (Crossley et al. 1966); which has been observed during the transition from the supine to upright posture (Nielsen et al. 1939), and during heating in an upright posture (Rowell et al. 1970). In the absence of other factors, such as exercising muscle activity or behavioural thermoregulation, there may be an intolerable accumulation of heat or development of pre-syncopal symptoms. Previous research (Ciuha et al. 2019; Fisher et al. 2022) has identified a regional variation in the skin blood flow response to these external stressors, suggesting that the competition between regulatory vascular mechanisms exerts specific and altered control on different regions of the body. Specifically, blood flow in the arms is predominantly under thermoregulatory control, whereas the blood flow in the legs is predominantly regulated by the baroreflex. Further research conducted by our group has postulated that in response to combined postural and heat stress, arm vasculature responds swiftly to input from multiple mechanisms (i.e. thermoregulatory and baroreflex), whereas the legs are more strongly innervated by the baroreflex alone (Fisher et al. 2022).

The current study tested the hypothesis that the separate and combined effects of artificial gravity (AG), hypoxia, and temperature will elicit differing regional responses in microvascular blood flow (M_BF_), and significantly alter haemodynamic control; specifically, that: (1) for the maintenance of blood pressure during AG, ambient temperature will most likely favour vasoconstriction of the peripheral circulation, particularly that of the lower limbs, (2) at higher ambient temperatures, the baroregulatory system will be somewhat diminished by the thermoregulatory response in the arms, via a withdrawal of vasoconstriction, (3) the combined vasodilatory effects of high ambient temperature and hypoxia, under a high gravitational gradient, will increase the rate of pre-syncopal symptoms.

## Materials and methods

The study was approved by the National Committee for Medical Ethics at the Ministry of Health of the Republic of Slovenia (approval no. 0120-180/2023/7) and conformed to the guidelines of the Declaration of Helsinki. The present study was conducted within the framework of a feasibility study for the European Space Agency BRAVE (Bed Rest, Artificial gravity and Vibration Exercise) study, during which subjects will be requested to conduct daily vibration resistance exercise on a short-arm human centrifuge. The present study was designed to establish the interaction of pressure and temperature regulation during passive exposure to high Gz loads in hypoxic cool and warm environments.

### Participant information

The minimum required sample size for investigating “repeated measures, within-between factors” was calculated using the results of a previous study (Ciuha et al. 2019). A difference of 4.9 °C in proximal–distal temperature gradient (ΔTsk_p-d_) during heating, and a difference of 3.0 °C during cooling, produced an effect size (d) between 1.58 and 3.38 for the association between temperature and microvascular blood flow. Assuming an α of 0.05 and β of 0.99, eight participants provide sufficient power to detect a statistical difference. Therefore, to account for any potential subject dropout, a total of ten male participants were recruited for the study. Their mean ± SD age was 27.9 ± 6.3 years, body mass 78.2 ± 10.3 kg, body stature 179.8 ± 6.3 cm, body mass index 24.2 ± 2.8 kg·m^2^, body surface area 2.0 ± 0.2 m^2^ (Mosteller 1987), and calculated blood volume 5.3 ± 0.5 L (Nadler et al. 1962). Prior to the start of the study, participants were familiarised with the study protocol and procedures and gave their written consent for participation. The following exclusion criteria were applied: smokers, physically inactive, diabetic, and/or a history of freezing or non-freezing cold injuries, cutaneous peripheral disease, or high or low blood pressure.

### Experimental protocol

Participants attended four testing sessions on different days separated by at least 24 h, in a repeated-measures, crossover design. For each participant, the experimental trials were conducted at the same time of the day in the Gravitational Physiology Laboratory, a European Space Agency ground-based research facility maintained by the Jožef Stefan Institute at the Nordic Centre Planica (Planica, Slovenia). The facility is located at an altitude of approximately 940 m (barometric pressure: 685 mmHg). The experimental trials were conducted on a short-arm human centrifuge (SAHC; Redwire, Belgium). The SAHC has two nacelles, each aligned on either side of the rotation axis. On one of the nacelles, participants are positioned supine in a cradle, with their head towards the axis of rotation and their feet outwards and positioned on a force platform. The other nacelle serves as a counterweight. Participants on the SAHC experience elevated gravitational acceleration in the head-to-foot direction. At maximum SAHC angular velocity, the maximum acceleration is equivalent to 4 Gz at the footplate. While this type of gravitational stimulus does not exactly replicate standing in normal gravity, the footward hydrostatic shift is similar.

**Fig. 1:**
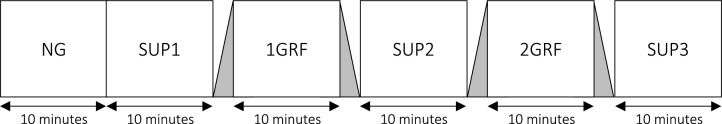
Session protocol displaying the order of conditions following the time for acclimation and instrumentation (not shown in figure), identical in each of the four sessions. Grey areas represent the 90-s ramp up/down for centrifugation

Figure 1 displays the protocol used in the present study, which was identical in each of the four testing sessions, as shown in Table 1. As indicated in Table 1, ambient temperature and partial pressure of oxygen (PO_2_, mmHg) were the key differences between trials. Relative humidity (%RH) remained constant at 49.5 ± 8.1%. The order of sessions was randomised via a Latin square design.

**Table 1:** Ambient conditions experienced in each session

	Warm	Cool
	*Warm normoxia (WN)*	*Cool normoxia (CN)*
Normoxia	PO_2_ = 133 mmHg Alt. = 940 m Ta = 29.1 ± 0.8 °C	PO_2_ = 133 mmHg Alt. = 940 m Ta = 18.4 ± 0.8 °C
Hypoxia	*Warm hypoxia (WH)* PO_2_ = 92 mmHg Eqv. Alt. = 4000 m Ta = 29.1 ± 0.8 °C	*Cool hypoxia (CH)* PO_2_ = 92 mmHg Eqv. Alt. = 4000 m Ta = 18.4 ± 0.8 °C

PO_2_  partial pressure of oxygen, *Eqv. Alt   * equivalent altitude, *Ta*   ambient temperature

Upon arrival at the laboratory, participants had their height and naked weight recorded, before donning shorts and a t-shirt without shoes/socks. Before entering the SAHC chamber, the participants rested in a thermoneutral ambient for 20-mins. They then entered the SAHC room and were fully instrumented, a further 10-mins of resting occurred, and maximum cutaneous blood flow responses were obtained (described in Sect. "Data collection methods"). With the exception of the first condition, in which the subjects were standing in normal gravity (NG), the remaining five conditions were conducted with the subject supine on the nacelle of the SAHC. During the trials, the subjects were either resting supine (SUP), or were exposed to a gravitational vector in the head-to-foot direction, by adjusting the angular velocity of the centrifuge. The definition of gravitational stimuli experienced by the participants was defined by the ground reaction force (GRF) as measured with a force platform situated at the feet. Measuring GRF rather than Gz is required for the production of a consistent acceleration profile between participants of differing body anthropometrics, due to the non-uniform acceleration produced by the SAHC. Table 2 provides mean ± SD of value the distance the head, heart, COM, and feet, from both the force platform and the SAHC centre of rotation. Centrifugation at 1GRF relates to 100% of participants bodyweight measured at the force platform (equivalent to 1Gz at the heart level), whilst 2GRF relates to 200% of the participants bodyweight measured on the force platform (equivalent to 2.0 Gz at the heart level). In the present study, 1GRF centrifugation produced 99.9 ± 9.8% of participants’ bodyweight (i.e. 1Gz) and 2GRF produced 187.0 ± 4.9% (i.e. 1.9 Gz) of bodyweight. 2GRF did not achieve 200% bodyweight due to limitations in the SAHC motor speed, which are in place for safety purposes.

**Table 2:** Mean ± SD calculated distances from force platform, and SAHC centre of rotation

	Eye	Heart	COM	Foot
Distance from the force platform (cm)	167.3 ± 5.7	143.6 ± 4.9	109.8 ± 3.7	0.0 ± 0.0
Distance from the centre of rotation (cm)	72.7 ± 5.7	96.4 ± 4.9	130.2 ± 3.2	240.0 ± 0.0

Calculated from average values presented in Paquette (2009). *COM*   centre of mass

The six experimental conditions, depicted in Fig. 1, were: (i) standing in normal gravity (NG), (ii) supine (SUP1), (iii) supine with the angular velocity of the centrifuge adjusted to maintain 1GRF on the force platform (1GRF), (iv) supine (SUP2), (v) supine with the angular velocity of the centrifuge adjusted to maintain 2GRF on the force platform (2GRF), (vi) supine (SUP3). During the trials, participants were requested to remain still with no movement in their right (instrumented) arm and leg, and without crossing their legs and/or arms. Subjects were monitored with video cameras, and were in constant audio contact with the SAHC controller. The test would have been stopped if any of the following indicators of pre-syncope were observed: persistent decrease in systolic blood pressure > 35 mmHg, or a decrease in heart rate > 15 b·min^−1^.

### Data collection methods

*Laser Doppler flowmetry (LDF)—*measured at the forearm (ArmBF) and calf (LegBF), on the right side of the body, as a non-invasive measure of capillary, arteriole, and venule perfusion (Moors Instruments Laser Doppler monitor, MoorVMS-LDF, Moor Instruments, UK). The device was calibrated using a fluid undergoing Brownian motion before each participant. Arbitrary units produced by the LDF (Laser Doppler units: LDU) were converted into cutaneous vascular conductance (CVC) as a ratio of LDU to mean arterial pressure. They were then normalised against maximum blood flow for both the arms and legs in normothermic conditions to produce a relative measure of percentage maximum blood flow (%maxBF). Maximum blood flow values were obtained by locally heating the limb and producing a post-occlusion reactive hyperaemia (PORH) via occlusion then release the of limb after 4 min.

*Cardiovascular –* Measurement of heart rate (HR, min^−1^), stroke volume (SV, mL), cardiac output (CO, L·min^−1^), systemic vascular resistance (SVR, mmHg⋅min⋅mL^−1^), rate pressure product (RPP, mmHg⋅min^−1^), systolic blood pressure (SBP, mmHg), diastolic blood pressure (DBP, mmHg), mean arterial pressure (MAP, mmHg), and oxygen saturation (SpO_2_, %) occurred continuously throughout the protocol (Finapres NOVA, Finapres Medical Systems B.V., Netherlands). Haemodynamic responses were calculated using the model flow algorithm (Wesseling et al. 1993) utilising the finger volume-clamp method and a five-lead electrocardiogram (ECG). Reconstructed systolic and diastolic blood pressures were calculated via direct finger pressure measurements using waveform filtering and level correction (Westerhof et al. 2002), and normalised to the heart level via a height correction unit measuring the hydrostatic pressure difference. Additionally, participants held their arm in a sling with the measured finger at the heart level throughout all conditions.

*Skin temperature (T*_*sk*_*) –* Skin temperatures were measured at minute intervals using wireless iButton thermistors (type DS1921H, Maxim/Dallas Semiconductor Corp., USA) located at four sites (mid-belly of the bicep brachii, pectoralis major at the mid-clavicular level, rectus femoris at the femur midpoint, gastrocnemius on the medial aspect) on the right side of the body. Weighed skin temperature (T_sk_) was then determined using Ramanathan (1964) equation.

*Gastrointestinal temperature (T*_*gi*_*)—*Deep body temperature was measured via ingested telemetric pills (BodyCap, eCelsius performance, Caen, France) measuring gastrointestinal temperature. The pills were ingested 60 min before start of the experimental protocol, and participants avoided drinking water after ingestion.

*Subjective measures –* Participants were requested to verbally provide their thermal sensation and thermal comfort every 5-min (ASHRAE 2017). Temperature sensation was measured from -3 (cold) through to 3 (warm), and thermal comfort was measured from 0 (comfortable) through to 3 (very uncomfortable). Finally, for participant safety, motion sickness was recorded during centrifugation using a 6-point scale ranging from 0 (no symptoms) to 6 (vomiting).

### Mechanistic analyses

*Heart rate variability (HRV) –* Heart rate was measured with a five-lead ECG, and the resultant R–R interval (RRi) was analysed via Kubios HRV Software (Version 3.5.0, Kubios Oy, Finland). Fast-Fourier transformation (FFT) frequency analysis of the RRi signal was conducted for each of the 10-min stages during the protocol, using a 5-min selection ending 1 min before the end of each interval and a medium beat correction threshold. The contributions of the parasympathetic (PNS) and sympathetic nervous systems (SNS) as an index, alongside a so-called ‘stress index’, were provided for each of the 10-min stages. In addition, the relative powers of each spectral frequency were calculated to represent the relative power of each component in proportion to the total power (Karim et al. 2011). Finally, the ratio of the absolute powers of the LF and HF bands (LF/HF ratio) was calculated, which is an important indicator of sympatho-vagal balance.

*LDF wavelet transform analysis (WTA)* WTA was shown to provide information regarding the regulation of skin perfusion occurring via interactions of autonomic stimulation, endothelial control, and myogenic activities (Bagno and Martini 2015; Bračič and Stefanovska 1998; Stefanovska et al. 1999). Prior spectral investigations of LDF signals using WTA have identified six frequency bands which relate to differing vascular control mechanisms: Band I (cardiac; 0.6–2.0 Hz), Band II (respiratory; 0.145–0.6 Hz), Band III (myogenic; 0.052–0.145 Hz), Band IV (neurogenic; 0.021–0.045 Hz), Band V (endothelial nitric oxide (NO) dependent; 0.0095–0.021 Hz), and Band VI (endothelial NO independent; 0.005–0.0095 Hz). In the present study, WTA analysis was conducted at each of the six protocol stages, using a 2-min stable window at minutes 3–5, to avoid unstable signals as a result of pre-syncopal symptoms.

### Statistical analyses

Following the conversion of raw LDF data into %maxBF data (described above), these data were averaged to produce minute values throughout the full protocol, presented as mean ± SD. Cardiovascular data, measured beat by beat, was also averaged to produce minute averages. Finally, *T*_sk_ and *T*_gi_ were averaged to produce minute values throughout the protocol. All data is presented as mean ± SD. Multiple three-way repeated measures ANOVA compared the effect of three independent variables—level of gravity (GRAV), temperature (TEMP), and PO_2_ (OXYGEN)—on the dependent variables (ArmBF, LegBF, HR, SV, CO, SVR, RPP, SYS, DIA, MAP, T_sk_, T_gi_). Data was analysed using IBM SPSS statistics (Version 26, IL, USA) and Graphpad (Graphpad Prism 9, Version 9.1.2, USA). An alpha value of *p* < 0.05 was considered to represent statistical significance.

## Results

All participants completed the NG and 1GRF conditions in all four conditions. No participant experienced full syncope under any test condition; however, during 2GRF several participants exhibited pre-syncopal symptoms resulting in premature termination of the trial under some of the environmental conditions. Time to cessation was 10.0 min for the cool normoxia (CN) trial, 9.1 ± 1.3 min for the cool hypoxia (CH), 8.8 ± 1.7 min for the warm normoxia (WN) trial, and 8.4 ± 2.0 min for the warm hypoxia (WH) trial. Therefore, no participant experienced early cessation of the trial in CN; however, early cessation was experienced in all other trials.

Participants reported being more thermally comfortable in NG (0.5 ± 0.2), compared to 1GRF (0.7 ± 0.4) and 2GRF (1.0 ± 0.1) across all conditions. Ratings of perceived exertion also increased with increasing G-load from 6.4 ± 0.3 during the NG trial to 7.0 ± 0.3 in the 1GRF and 8.7 ± 0.3 in the 2GRF trials.

### Microvascular blood flow

**Fig. 2:**
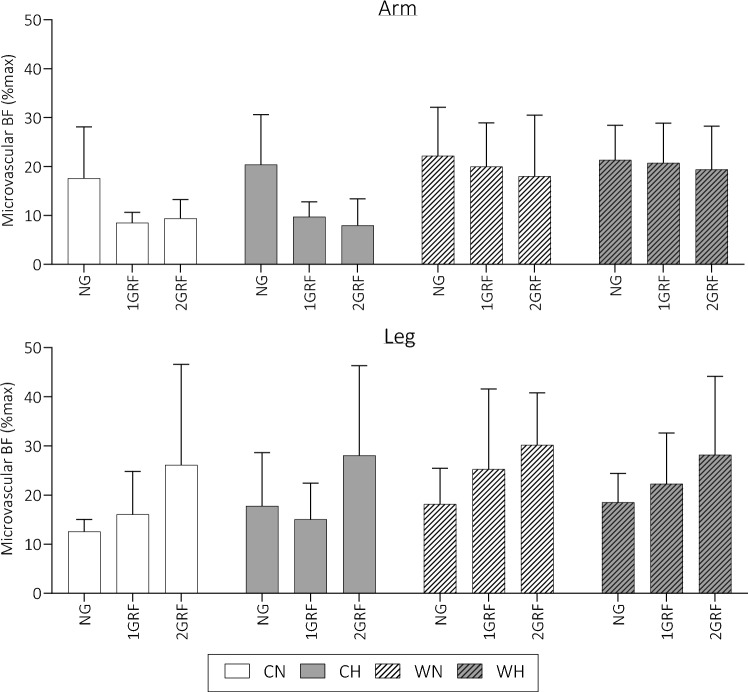
Mean ± SD microvascular blood flow in differing ambient conditions and gravitational loads. *CN* cool normoxia, *CH* cool hypoxia, *WN* warm normoxia, *WH* warm hypoxia

Figure 2 presents responses of microvascular blood flow in the different ambient conditions and gravitational loads. ArmBF was significantly reduced as a result of GRAV (*p* < 0.001, F = 21.11). Multiple comparisons analysis identified that the significant effect of GRAV was a result of the differences between NG (20.7%max) and 1GRF (14.9%max, *p* < 0.001), and between NG and 2GRF (13.8%max, *p* < 0.001). The difference between 1 and 2GRF was negligible (1.1%max, *p* = 0.624). Additionally, TEMP caused a significant increase in armBF, regardless of GRAV and OXYGEN, increasing from 12.25%max in cool conditions to 20.26%max in warm conditions (*p* < 0.001, F = 23.47).

LegBF, however, was only significantly affected by GRAV (*p* < 0.001, F = 13.64). Unlike armBF, the multiple comparisons analyses identified that the difference between NG and 1GRF was non-significant, whereas the 2GRF condition (27.4%max) produced significantly higher legBF than both NG (16.8%max, *p* < 0.001) and 1GRF (19.7%max, *p* < 0.001).

### Skin and deep body temperature

**Fig. 3:**
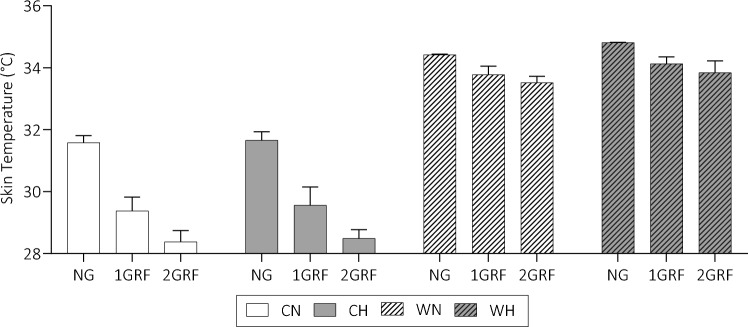
Skin temperatures under differing ambient conditions and gravitational loads. *CN* cool normoxia, *CH* cool hypoxia, *WN* warm normoxia, *WH* warm hypoxia

Figure 3 presents mean ± SD skin temperature in all conditions. The NG condition produced the highest average T_sk_ (33.1 °C) compared to 1GRF and 2GRF, which were similar (1GRF = 31.7 °C, 2GRF = 31.1 °C; *p* < 0.001, F = 450.0). T_sk_ was considerably higher in the warm condition compared to the cool (cool = 29.9 °C, warm = 34.1 °C; *p* < 0.001, F = 5481). In addition, the hypoxic condition produced a higher *T*_sk_ than the normoxic condition (Nor = 32.1 °C, Hyp = 33.5 °C; *p* < 0.001, F = 17.30). Finally, the interactions of TEMP + GRAV (*p* < 0.001, F = 133.0) and of TEMP + OXYGEN (*p* = 0.047, F = 4.029) on *T*_sk_ were significant. For the interaction of TEMP + GRAV, the drop in T_sk_ as a result of the GRAV was significantly larger in cool conditions ( – 3.2 °C) than warm conditions ( – 0.9 °C) (*p* < 0.001). The difference between cool and warm conditions in hypoxia (4.4 °C) was significantly higher than the difference in normoxic conditions (4.1 °C) (*p* < 0.001).

There was no difference in any of the measured deep body temperatures as a result of GRAV, TEMP, OR OXYGEN. The mean values were CN = 37.1 ± 0.2 °C, CH = 37.2 ± 0.2 °C, WN = 37.1 ± 0.2 °C, and WH = 37.2 ± 0.2 °C.

### Haemodynamics

Table 3 displays the haemodynamic responses for each gravitational stimulus.

HR significantly increased by ~ 25 min^−1^ from NG to 2GRF, as a result of GRAV (*p* < 0.001, F = 22.81). A significantly higher HR was observed in the warm condition (cool = 78.9 min^−1^, warm = 88.4 min^−1^; *p* = 0.006, F = 7.798) and in the hypoxic condition compared to normoxia (Nor = 81.5 min^−1^, Hyp = 85.9 min^−1^; *p* = 0.02, F = 5.613).

SV was significantly reduced by the increase in GRAV (*p* < 0.001, F = 11.91), with the multiple comparisons analysis identifying that SV was significantly lower in 2GRF compared to both NG ( – 15.4 mL, *p* < 0.001) and 1GRF ( – 14.8 mL, *p* < 0.001).

RPP significantly increased as a result of increases in GRAV (*p* < 0.001, F = 22.00), and by higher TEMP (*p* = 0.021, F = 5.458). Multiple comparisons analysis identified that RPP was significantly higher in 2GRF, compared to both NG (+ 3459.0 mmHg·min^−1^, *p* < 0.001) and 1GRF (+ 3519.0 mmHg·min^−1^, *p* < 0.001). Additionally, the cool conditions (12,426.3 mmHg·min^−1^) produced higher RPP values than the warm conditions (11,267.7 mmHg·min^−1^).

SVR was significantly higher at greater GRAV (*p* = 0.003, F = 6.324), and lowered by the increase in TEMP (cool = 1.4 mmHg·min·mL^−1^, warm = 1.1 mmHg·min·mL^−1^; *p* = 0.001, F = 11.44). Multiple comparisons highlighted the difference between NG vs. 2GRF (+ 0.3 mmHg·min·mL^−1^, *p* = 0.002) as a significant contributor to this difference.

SBP was lowest in NG (129.9 mmHg) compared to both 1GRF (+ 11.0 mmHg) and 2GRF (+ 16.4 mmHg) (*p* < 0.001, F = 13.07). Additionally, the warm condition caused a significantly lowered SBP compared to the cool (cool = 144.2 mmHg, warm = 137.1 mmHg; *p* < 0.001, F = 11.20).

DBP was also significantly elevated by GRAV (*p* < 0.001, F = 20.92). Multiple comparison analysis identified elevated DAP in the 2GRF condition (107.1 ± 14.9 mmHg), which displayed significantly higher values than either NG ( – 22.7 mmHg, *p* < 0.001) or 1GRF ( – 15.9 mmHg, *p* < 0.001).

SpO_2_ was significantly higher in the 2GRF condition (91. 8 ± 6.5%) compared to either NG (90.9 ± 5.2%) or 1GRF (90.9 ± 6.2%) (*p* = 0.022, F = 3.970). The higher ambient TEMP caused a significantly lower SpO_2_ (cool = 91.8 ± 1.0%, warm = 90.5 ± 0.6; *p* < 0.001, F = 12.16). SpO_2_ in the normoxic conditions was 96.4 ± 1.1% and in the hypoxic conditions 86.1 ± 1.3% (*p* < 0.001, F = 659.2).

**Table 3:** Mean ± SD haemodynamic values at each ambient condition and gravitational stimulus

Haemodynamics	Sig	Condition	NG	1GRF	2GRF
Heart rate *(min*^*−1*^*)*	a,b,c	CN	73.1 ± 11.7	66.5 ± 12.0	84.8 ± 14.6
		CH	79.2 ± 8.2	74.3 ± 12.0	95.5 ± 14.7
		WN	80.7 ± 7.2	76.5 ± 7.1	107.1 ± 21.0
		WH	87.9 ± 10.8	80.6 ± 11.2	97.5 ± 22.6
Stroke volume *(mL)*	a	CN	76.1 ± 11.3	83.6 ± 16.1	57.9 ± 15.9
		CH	77.2 ± 21	73.7 ± 16.9	61.1 ± 17.9
		WN	73.0 ± 10.4	71.6 ± 11.9	60.8 ± 13.1
		WH	71.5 ± 11	66.4 ± 10.8	61.7 ± 18.4
Cardiac output *(L·min*^*−1*^*)*		CN	5.5 ± 1.2	5.6 ± 1.6	5.1 ± 1.8
		CH	6.0 ± 1.4	5.6 ± 1.5	5.7 ± 1.9
		WN	5.8 ± 0.8	5.4 ± 0.8	6.5 ± 1.8
		WH	6.1 ± 0.9	5.3 ± 0.8	6.3 ± 1.7
Rate pressure product *(mmHg·min*^*−1*^*)*	a,b	CN	10,452.6 ± 1803.7	10,382.6 ± 1557.7	15,031.2 ± 2450.5
		CH	11,613.3 ± 1959.3	12,127.6 ± 2801.9	15,871.6 ± 4289.4
		WN	10,181.6 ± 960.2	9840.0 ± 1316.5	16,160.3 ± 3572.9
		WH	10,608.1 ± 2307.2	10,266.6 ± 1233.6	14,551.6 ± 3215.7
Systemic vascular resistance *(mmHg·min·mL*^*−1*^*)*	a,b	CN	1.1 ± 0.2	1.3 ± 0.4	1.7 ± 0.5
		CH	1.1 ± 0.3	1.3 ± 0.3	1.4 ± 0.6
		WN	1.0 ± 0.2	1.1 ± 0.3	1.1 ± 0.3
		WH	0.9 ± 0.2	1.1 ± 0.3	1.1 ± 0.4
Systolic pressure *(mmHg)*	a,b	CN	132.9 ± 11.1	145.8 ± 11.8	161.3 ± 11.2
		CH	126.7 ± 8.4	148.6 ± 13	149.8 ± 16.1
		WN	131.3 ± 10.8	133.5 ± 14.9	150.2 ± 17.7
		WH	128.6 ± 17.3	135.7 ± 14.9	143.0 ± 15.0
Diastolic pressure *(mmHg)*	a	CN	85.1 ± 10.2	92.6 ± 9.8	115.4 ± 10.7
		CH	80.6 ± 9.6	91.7 ± 11.9	102 ± 14.5
		WN	85.2 ± 10.2	87.7 ± 13.3	106.4 ± 15.5
		WH	86.7 ± 16.8	92.8 ± 13.1	103.5 ± 15.5
Mean arterial pressure *(mmHg)*	a	CN	103.8 ± 13.9	115.0 ± 15.0	133.7 ± 14.1
		CH	97.7 ± 9.1	112.8 ± 25.7	123.1 ± 21.0
		WN	103.0 ± 12.6	105.7 ± 17.3	123.3 ± 21.4
		WH	102.7 ± 18.8	109.5 ± 19.2	118.6 ± 16.1
Oxygen saturation (%)	a,b,c	CN	95.9 ± 1.5	97.3 ± 1.1	98.1 ± 1.5
		CH	86.5 ± 1.8	85.4 ± 3.4	87.2 ± 4.9
		WN	95.5 ± 1.2	95.9 ± 1.0	96.3 ± 1.4
		WH	85.2 ± 2.2	84.9 ± 2.3	84.8 ± 3.3

*CN*   cool normoxia, *CH*   cool hypoxia, *WN*   warm normoxia, *WH*   warm hypoxia, *a*   significant main effect of gravity, *b*  significant main effect of temperature, *c*  significant main effect of oxygen, *p* < 0.05

### Mechanistic analyses

**Fig. 4:**
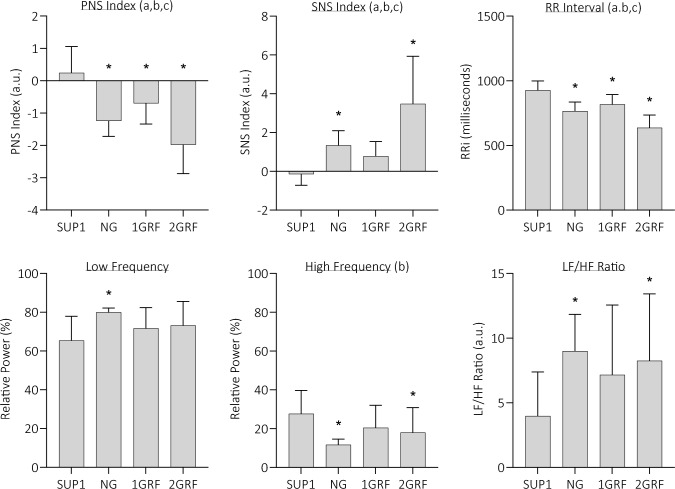
Heart rate variability indices as mean of all four conditions. *PNS* parasympathetic nervous system, *SNS* sympathetic nervous system, *LF* low frequency, *HF* high frequency, *a* significant effect of gravity, *b* significant effect of temperature, *c* significant effect of oxygen, * significantly differently to SUP1, *p* < 0.05

*Heart rate variability* Fig. 4 displays the HRV values averaged from each GRAV condition; however, analyses assessing the effects of TEMP and OXYGEN were also conducted. The single main effect of GRAV caused significant differences between conditions in PNS index (*p* < 0.001, F = 23.02), SNS index (*p* < 0.001, F = 14.49), and RRi (*p* < 0.001, F = 49.24). In all of these variables, the 1GRF condition exhibited the smallest deviation from SUP1 (i.e. rest period); however, these differences were still significant in both PNS index (difference = 0.9 a.u.; *p* < 0.001) and RRi (difference = 109 ms; *p* < 0.001). 2GRF resulted in the greatest sympathetic autonomic strain and was thus significantly higher in the SNS index (NG = 1.3, 1GRF = 0.8, 2GRF = 3.6; *p* < 0.001) and significantly lower in the PNS index (NG =  – 1.2, 1GRF =  – 0.7, 2GRF =  – 2.1; *p* < 0.001) and RRi (NG = 765.8 ms, 1GRF = 818.3 ms, 2GRF = 628.5 ms; *p* < 0.001), in both other GRAV conditions.

The TEMP condition as a single main effect caused PNS index (cool = 1.0 ± 0.8, warm =  – 1.7 ± 0.7, *p* < 0.001, F = 14.49), RRi (cool = 777.9 ± 110.9 ms, warm = 700.5 ± 97.2 ms, *p* < 0.001, F = 14.49), and relative HF (cool = 1.0 ± 0.8, warm =  – 1.7 ± 0.7, *p* < 0.001, F = 14.49) to be significantly lower in the warm condition compared to cool. In contrast, SNS index (cool = 1.0 ± 0.8, warm = 1.7 ± 0.7, *p* < 0.001, F = 14.49) was significantly higher in warm ambient conditions, regardless of the GRAV condition.

Similarly to TEMP, OXYGEN also elicited higher sympathetic activity when under hypoxic conditions. There were significant decreases in PNS index (normoxia = 1.1 ± 0.6, hypoxia = 1.6 ± 1.5, *p* < 0.001, F = 14.49), and RRi (normoxia = 781.0 ± 89.4 ms, hypoxia = 697.4 ± 102.3, *p* < 0.001, F = 14.49), whilst SNS significantly increased (normoxia = 1.4 ± 0.7, hypoxia = 2.0 ± 1.2, *p* < 0.001, F = 14.49).

**Fig. 5:**
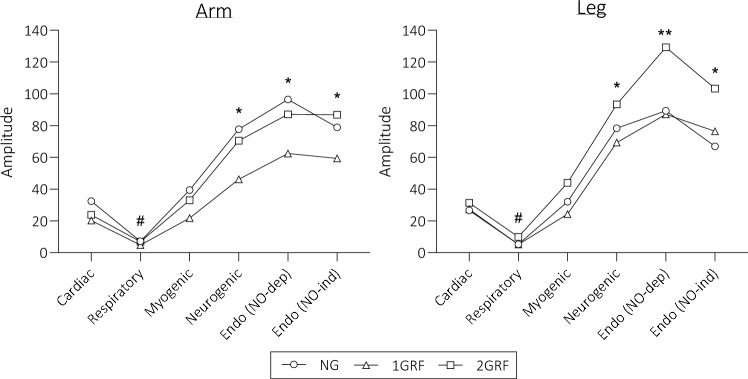
Wavelet transform analyses averaged across all TEMP and GRAV conditions. *Frequency band value significantly higher than that of the first three bands in all GRAV conditions, **Frequency band value significantly higher than that of all other bands in all GRAV conditions, #Frequency band significantly lower than that of all other bands in all GRAV conditions

*Wavelet transform analysis –* Initial analyses assessing the effects of ambient conditions (i.e. main effects of TEMP and OXYGEN) identified no differences in WTA amplitude at any frequency band or GRAV condition; therefore, further analyses only assessed differences between the mean amplitudes of all ambient conditions at each GRAV load (Fig. 5). These analyses identified significant effects of both frequency band (*p* < 0.001, F = 70.31) and GRAV (*p* = 0.32, F = 3.673), however, there was no interaction effect of these variables.

Follow-up multiple comparisons analyses were conducted, and three main observations were made. Frequency bands 4 (neurogenic), 5 (endothelial NO-dependent), and 6 (endothelial NO-independent) were all significantly higher than bands 1 (cardiac), 2 (respiratory), and 3 (myogenic) (all: *p* < 0.001).

In the arm, these frequency bands did not display amplitudes that were different from each other. However, in the legs, the endothelial (NO dependent) frequency band was shown to be significantly higher than any other variable in each of the GRAV conditions (*p* < 0.001). Secondly, the frequency band representing respiratory control (band 2) displayed significantly lower amplitude (and therefore vascular control) than other frequency bands in both the arm and leg (*p* < 0.001). Finally, the cardiac and myogenic frequency bands (bands 1 and 3) displayed similar amplitudes and were therefore not statistically different (*p* > 0.05), in both the arm and leg regions; however, the amplitude of these bands was still significantly lower than that of bands 4, 5, and 6.

## Discussion

The main findings of the current study are that SkBF in the leg (i.e. legBF) was unaffected by the varying ambient conditions and only significantly responded to the change in GRAV. However, no difference was observed between NG and 1GRF, whilst it was clear that 2GRF centrifugation in the head-to-foot direction was sufficient to overcome the baroreflex and allow pooling of blood in the lower limbs. ArmBF, however, responded significantly to both the thermal and gravitational stressors. This is in line with previous observations (Ciuha et al. 2019; Fisher et al. 2022) purporting that the arms respond to the interaction of multiple regulatory vascular mechanisms, while the legs do not. Despite the aforementioned vascular mechanisms, the effects of both ambient stressors (TEMP and OXYGEN) caused a 16% reduction in the time to test cessation from CN to WH conditions, based on indicators of pre-syncope, during the 2GRF condition. This is likely due, in part, to the significant pooling of blood observed in the legBF in the 2GRF condition. Interestingly, no effect of hypoxia was observed in either armBF or legBF, but there was an increase in HR and an expected decrease in SpO_2_; yet, a significant decrease in the time to test cessation due to hypoxia occurred.

### Orthostatic tolerance (OT)

Time-to-test cessation at 2GRF represents the influence that ambient conditions place on the cardiovascular system’s ability to continuously supply oxygenated blood to the brain and other vital organs, supported by the sudden drop in HR and MBP used as a primary indicator of syncope. Both TEMP and OXYGEN caused separate and combined reductions in OT, leading to frequent early cessation of the 2GRF phase due to pre-syncopal symptoms. In fact, all participants were able to complete the full 2GRF phase in the CN condition, whereas the overall decrease as a result of the high ambient temperatures and hypoxia was 9.5% and 7.4%, respectively. These results indicate that cool temperatures are the most suitable operating temperatures for passive centrifugation, with the potential for exercise during centrifugation on an SAHC (Diaz et al. 2015; Duda et al. 2012; Katayama et al. 2004; Kramer et al. 2020a, b). Considerations should be given to define the most suitable operating temperature for different exercise types (i.e. aerobic, resistance, etc.). Therefore, further research must extend the scope of SAHC utilisation beyond simple exercise modalities and consider the effect of ambient conditions and their subsequent role in exercise performance; similar to terrestrial research, yet considering the unique aspects of an SAHC. The negative effects of temperature on OT are well documented (Schlader et al. 2016), by nature of individual and interactive mechanisms, including central cardiac responses, arterial and venous changes, redistribution of blood, and autonomic responses. In contrast, the literature regarding the effects of acute hypoxia on OT is sparse (Blaber et al. 2003; Halliwill and Minson 2005), but alludes to altered autonomic activity and vasodilatation of the splanchnic circulation as triggers for pre-syncope. However, it is known that in response to an acute hypoxic stimulus, different regional vascular responses are initiated. There is vasodilation of peripheral non-acral (Minson 2003) and splanchnic (Halliwill and Minson 2005; Rowell and Blackmon 1986) vasculature, concomitant with vasoconstriction of the acral (Jones et al. 2021) and pulmonary (Weir and Archer 1995) vasculature. Further, Ainslie et al. (2007) reported that during exposure to normobaric hypoxia equivalent to an air altitude of 4500 m, vasodilation of cerebral arteries through cerebral autoregulation allows stable oxygenated blood supply to the brain. However, the frequent occurrence of pre-syncopal symptoms in the current study suggests that the observations of Ainslie et al. (2007) at 4500 m do not apply to the current study; if, in fact, the cause of pre-syncope is a result of unstable oxygen delivery to the brain caused by competition for blood flow pooling in the lower extremities. The combination of these aforementioned factors, when combined with hypergravity causing lower limb blood pooling, would indicate reduced blood supply to the upper regions of the body and particularly the brain.

### Microvascular blood flow response

The orthostatic intolerance described in the previous section is the result of varied and profound cardiovascular responses to the temperature, gravity, and hypoxic stimuli. ArmBF was significantly affected by both the ambient temperature and head-to-foot gravitational force. As mentioned, these results match previous observations whereby the arm vasculature is controlled by interactions of multiple vasomotor mechanisms when under thermal and gravitational stressors. Interestingly, additional analyses identified that AG (i.e. 1GRF and 2GRF) produced significantly lowered armBF than in the NG condition, primarily in the cool conditions. Conversely, there was an attenuated drop in armBF during the warm conditions, which may have contributed to the higher incidence in pre-syncopal symptoms. Increasing blood pooling in the skin vascular bed, combined with relaxation of cutaneous veins may be responsible for a drop in MAP of 5–10 mmHg (Crandall et al. 1999); in addition to the gravitational challenge, this would certainly impact orthostatic tolerance. It is also possible that this response is due to the additional convective heat loss mechanism which would be more prominent during centrifugation. During the 2GRF trial, participants are spinning at an equivalent speed of 27.7 km/hour, which induces a wind speed of 1.4 m·s^−1^ of the head and 4.2 m·s^−1^ at the foot. A convective mechanism of this magnitude will certainly induce reduced *T*_sk_ and consequently initiate vasoconstriction, which will reduce SkBF, when compared to standing in still air alone.

LegBF, however, was only significantly affected at 2GRF. Also, in contrast to the armBF, similarities were observed between NG and 1GRF exposures, whereas the 2GRF condition produced the highest legBF (~ 7–10%max higher). These results match those of Verma et al. (2018) and Goswami et al. (2015), who identified similarities in cardiovascular responses to 1 Gz (heart level) centrifugation and standing. Goswami et al. (2015) also identified increases in calf venous volume, particularly during 2 Gz at the foot level. During hypergravity exposure, lower body microvasculature experiences high transmural pressures from both the arterial and venous branches (Habazettl et al. 2016), which is largely mediated by autonomic control and myogenic vasoconstrictor activities. Beyond pressure limits of myogenic perfusion control at ~ 150 mmHg (Bullivant 1978; Strandgaard et al. 1974), expected during centrifugation, resistance vessels may fail to overcome the transmural pressure leading to excess lower limb pooling of blood as much as 700–800 mL (Bradley and Davis 2003; Lipsitz 1989). Interestingly, in contrast to Habazettl et al. (2016), who noted that vascular resistance was maintained even at 2 Gz (foot level), in the present study there was a significantly higher microvascular flow at 2GRF vs other conditions; these disparities may be due to the length of exposure between the two studies (4-min vs. 10-min). In contrast to the findings of the present study, Watenpaugh et al. (2004) noted altered responses of thigh and calf skin blood flow measured by LDF, proposing that decreases in blood flow occurred during transient changes in centrifugation up to 1 Gz at the heart level. It is possible that the differences in length (~ 40 s) and magnitude (1.0 Gz foot level or 0.4 Gz heart level) of centrifugation by Watenpaugh et al. (2004) were sufficient to produce significantly different observations from the present study. As previously mentioned, there was no significant response of either armBF or legBF to the hypoxic condition, which is perhaps unusual as non-acral skin would be expected to observe an element of vasodilation (Minson 2003).

### Haemodynamic response

During standing and 1GRF trials, haemodynamic responses did not significantly differ from each other. It is therefore likely that the 1GRF stimulus is suitable at recreating the hydrostatic and cardiovascular strain associated with standing. These results clearly match those of previous research, in relation to autonomic and blood pressure indices (Verma et al. 2018), and cardiovascular and cerebral responses (Goswami et al. 2015; Laing et al. 2020). This production of an equivalent AG stimulus to standing further promotes the use of centrifugation, at lower Gz, for the maintenance of cardiovascular health in situations where orthostatic stress cannot be regularly provided, such as the micro-gravity experienced during spaceflight. The 2GRF condition, however, produced a gravitational stimulus that, in certain conditions and for some participants, was intolerable for the full duration of the protocol phase (i.e. 10 min). In fact, across all ambient conditions the average tolerance was 91% of the full 10-min session (i.e. 9.1 ± 1.3 min). The haemodynamic responses reveal that until the point at which pre-syncope or the end of the test was reached, participants exhibited higher HR, BP, and RPP, concomitant with a lower SV. Even at 2GRF, these values would somewhat match the type of response expected during low intensity exercise (Laughlin 1999), except for the decrease in SV, despite the passive nature of centrifugation in the present experiment. In normal low-intensity exercise, SV would be expected to increase slightly rather than decrease, to compensate for the required increase in CO. The supine position would have limited diastolic filling (Warburton and Gledhill 2008), despite the centrifugal stimulus. Due to the passive nature of the protocol used in the present study, there was no additional venous return stimulated via exercise; capable of increasing venous return from the lower limbs via compression of veins during skeletal muscle contraction, sympathetic stimulation of vein walls, and skeletal muscle arteriole dilation (Berlin and Bakker 2014; Miller et al. 2005). Interestingly, while SpO_2_ was predominantly influenced by the hypoxic environment (82% of ANOVA variation), it was also significantly affected by TEMP and GRAV. SpO_2_ appeared to be ~ 1% higher in warm conditions, and in 2GRF, vs other conditions. Under these conditions the body is under greater physiological strain, and therefore produces a subsequent cardio-respiratory response; thereby improving oxygen delivery and subsequently SpO_2_.

The effects of heat stress via passive heating alone on haemodynamic responses are well known, including elevated skin and core temperatures, elevations in SkBF, increased HR and CO, and little to minimal change in MBP (Crandall and Gonzalez‐Alonso 2010). Besides the additional artificial gravitational stress, the current study results are in line with the literature whereby the ambient temperature had a significant effect on a number of variables; in particular, increases in HR, RPP, SVR, and SYS.

### Central and peripheral autonomic control

Measures of central autonomic function were achieved via HRV frequency-based analyses for detection of para/sympathetic regulation. As expected, the gravitational stressor produced large deviations from a supine baseline value (i.e. SUP1 condition) in HRV variables of SNS index, PNS index, and RRi, though not in the high- or low-frequency power bands. In agreement with the haemodynamic responses, NG and 1GRF produced similar responses in these variables (PNS, SNS, RRi), though 1GRF produced the lowest autonomic activity; in fact, SNS index during 1GRF was not significantly higher than that in the supine rest period. These similarities have been previously noted, yet increases in low-/high-frequency power distributions at 2 Gz at the foot in Verma et al. (2018) do not match those of the present study. The 2GRF centrifugation drew significantly lower PNS index and RRi, whilst SNS was significantly higher, which reveals the level of sympathetic autonomic response required to tolerate double Earth’s gravity for a sustained period. Indeed, while gravitational stress had a considerable effect on autonomic control, both ambient temperature and oxygen availability significantly impacted PNS index, SNS index, and RRi also. The interrelation of 2GRF, warm temperature, and hypoxia produced the greatest sympathetic drive, which was to be expected considering the singular effect each variable has on autonomic activity (Buchheit et al. 2004; Sollers et al. 2002; Stauss 2003).

Whereas central autonomic mechanisms were significantly affected by the main independent variables of ambient temperature and oxygen concentration; these had no effect on the peripheral mechanisms as indicated by spectral analyses of LDF signals using WTA. In fact, only the gravitational stimulus produced variation in the WTA results, as will be discussed with reference to each limb separately at each gravitational load (i.e. NG, 1GRF, 2GRF). The main finding was that significantly higher amplitudes were identified in bands 4, 5, and 6 in both the arm and leg, representing neurogenic, endothelial (NO -dependent), and endothelial (NO independent) (Bagno and Martini 2015) activities, respectively. Neurogenic activity represents the local influence of sympathetic control on the microvasculature. As described previously, the separate and combined effects of each independent variable in the present study caused increased central sympathetic drive, which was subsequently identified in the efferent pathways. Utilisation of endothelium-dependent and -independent vasodilators (acetylcholine and sodium nitroprusside, respectively) reveals the contribution of NO and prostaglandin E synthesis in band 5, whilst band 6 may be associated with other endothelial mechanisms such as endothelial-derived hyperpolarisation factor (Hodges and Cheung 2020; Kvandal et al. 2006, 2003). Each of these mechanisms are responsible for potent vasodilation; however, the lack of a severe drop in MAP and syncope at the haemodynamic and WTA time point suggests this level of vasodilation is unlikely. It may therefore be possible that instead of detection of the endothelial vasodilative mechanisms, the analysis may have detected the pooling of blood in peripheral regions due to excess transmural pressure. In addition, the leg in 2GRF produced an amplitude in band 5 (endothelial NO dependent) that was higher than any other band and any other condition, further exhibiting a potential error. To the authors’ knowledge, this is the first study which assesses WTA in individuals undertaking hypergravity procedures; more research is required to conclusively understand the potential error observed. It was also interesting that band 2 (respiratory) produced a negligible response in any condition, which may be due to the passive nature of the centrifugation; indeed, had aerobic or resistance exercise been conducted this may be different.

### Limitations and future perspectives

Whilst the comparison between warm and cool ambient conditions provides a suitable understanding of the upper and lower limits of thermoregulation, it may have been beneficial to compare these responses by means of a variation from a normothermic control. Studies have assessed the cardiovascular responses to SAHC, in which a normothermic environment of 22–23 °C was reported (Goswami et al. 2015; Watenpaugh et al. 2004). The aim of the present study was not to repeat these data, but build on the dataset by utilising adverse ambient conditions to elicit differing stress on the human body to further tease out the underlying mechanisms of thermoregulation and blood pressure control of the limbs.

Monitoring of microvascular circulation via LDF suitably presents responses of vasomotor mechanisms to alter peripheral vasculature. In addition, the transmural pressures generated by arterial and venous branches on capillaries during hypergravity may further indicate the stress elicited on the whole vascular system, rather than the microvasculature alone. However, to further understand the holistic influence of a, SAHC on blood flow and haemodynamic responses, an assessment of whole-body fluid shifts would be beneficial. Studies assessing the effects of SAHC on fluid shift within the body have been previously conducted. Takhtobina et al. (2020) detected changes in impedance throughout the body, with an overall redistribution of blood towards the foot, citing it as an effective predictor of circulation changes. Both Vaitl et al. (2002) and Howarth et al. (2008) noted significant decreases in thoracic fluid volumes as a result of centrifugation, stating that this would impact the perception of posture in the absence of vestibular influences and detect venous return, therefore, predicting presyncope, respectively. It is clear that segmental impedance would be a useful tool in cardiovascular monitoring and presyncope avoidance for SAHC operation.

Muscle activation during higher levels of AG may have altered the sympathetic response to ambient conditions, making the 2GRF condition, particularly, more tolerable by aiding the return of blood through muscle pump actions. Indeed, the AGBRESA study, which utilised continuous or intermittent 1 Gz centre of mass centrifugation for 30 min/day over 60 days, found large individual variability in muscle activation ranging from 1 to 96% of maximal voluntary contraction in the leg muscles (Kramer et al. 2020a, b). Therefore comparing AG to other forms of orthostatic strain (head-up tilt, lower-body negative pressure) would be difficult due to their more passive nature. In the present study, however, cardiovascular responses were only compared within participant in different AG and ambient conditions, decreasing the impact of individual variability of muscle activation.

Finally, a consideration should be made for use of an SAHC vs a long-arm human centrifuge (LAHC). In the present study, an SAHC was deemed more appropriate for two reasons. Firstly, the SAHC produces an acceleration gradient that acts in a head-to-foot direction, producing a leg-ward shift in fluid similar to that of lower-body negative pressure. Therefore the non-uniform acceleration of the SAHC provides a greater baroreflex stimulus for both high- and low-pressure baroreceptors located within the carotid sinus/aortic arch and the atria/pulmonary veins, respectively. As the aim of the study was to assess the interaction of baroreflex and thermoregulatory mechanisms in varying ambient conditions, this baroreflex activation was more appropriate. Secondly, while the primary aim of the study was to assess the mechanisms associated with cardiovascular control, a secondary aim was the assessment of haemodynamic responses to centrifugation via an SAHC due to its potential role as a spaceflight countermeasure.

The development of exercise countermeasures for future deep space missions will be aided by development of algorithms allowing the prediction of cardiovascular exercise responses in a variety of anticipated ambient conditions within future space vehicle and planetary habitats. For this, the development of such algorithms, as outlined by Bonjour et al. (2011) for exercise conducted on LAHC would need to be repeated for exercise conducted on SAHC, considering the non-uniform application of gravitational loading.

## Conclusion

To conclude, the present study demonstrates that human tolerance during passive centrifugation to 2GRF was significantly affected by the ambient conditions. Haemodynamic and lower limb blood flow responses, particularly in the higher temperature and Gz conditions, revealed a clear cardiovascular challenge which commonly led to pre-syncopal symptoms. Meanwhile, cool temperatures appeared to produce tolerable conditions in which participants may benefit from longer passive or active SAHC sessions if conducted regularly.

## Data Availability

Data available on request.
